# Articulated dental cast analysis of asymptomatic and symptomatic populations

**DOI:** 10.1038/ijos.2015.44

**Published:** 2016-04-01

**Authors:** Frank E Cordray

**Affiliations:** 1 Department of Orthodontics, College of Dentistry, Ohio State University, Columbus, USA

**Keywords:** dental arch displacement, condylar displacement, seated condylar position, intercuspal position, musculo-skeletal dysfunction, temporo-mandibular dysfunction, common muscle contraction headache

## Abstract

Dental instrumentation has long provided insight into the mechanism of musculo-skeletal function of the gnathic system. While large population studies associate dental arch displacement (DAD), especially laterally, with symptoms, mandibular condyle displacement (CD) resulting from DAD has not been targeted as possibly etiologic in the production of common muscle contraction headache (CMCH) and temporo-mandibular dysfunction (TMD). The objective was to evaluate the three-dimensional nature of DAD and CD between the seated condylar position (SCP) and the intercuspal position (ICP) and to compare results derived from large deprogrammed asymptomatic and symptomatic populations. A total of 1 192 sets of dental casts collected from asymptomatic and symptomatic populations were articulated in the SCP. The initial occlusal contact, DAD, and condylar displacement were evaluated for frequency, direction, and magnitude of displacement between the SCP and ICP. The data revealed significant displacement between the SCP and ICP of the condyles (displaced most frequently inferior (down) and posterior (distal)) and substantially increased frequency and magnitude of displacement of the dental arches (with posterior premature occlusal contacts, increased overjet, decreased overbite, midline differences, and occlusal classification changes) in symptomatic subjects. These discrepancies were statistically significant and clinically significant. The data support the concept of increased DAD and CD with dysfunction. Transverse condylar displacement, commonly presenting with dental cross bite, may be associated with CMCH and TMD. Displacement of the mandibular condyle may be an etiologic factor in CMCH and dysfunction of the temporo-mandibular joint.

## Introduction

Upon concluding his 1973 investigation comparing mandibular condyle position in the seated position (SCP) and the intercuspal position (ICP), Hoffman states: “When suitable studies are performed in the future, it will be interesting to learn whether the (ICP-SCP) distance is statistically different in healthy individuals and in those with pathology of the temporo-mandibular (TM) joint, periodontium, or occlusal surfaces of the teeth.^1^ This series of investigations^2^ (with Part I) is an attempt to answer this question.

The positional difference of the mandibular condyle between the SCP and ICP is a source of contention and conflicting judgments by those who rehabilitate dental occlusions. The difference is associated with contradictory theories of jaw movement, jaw recordings of positional and dynamic states, and the selection of dental instrumentation (articulators) for diagnosis and dental rehabilitation. It is, therefore, an important topic for further study and greater understanding.

**Figure 1:**
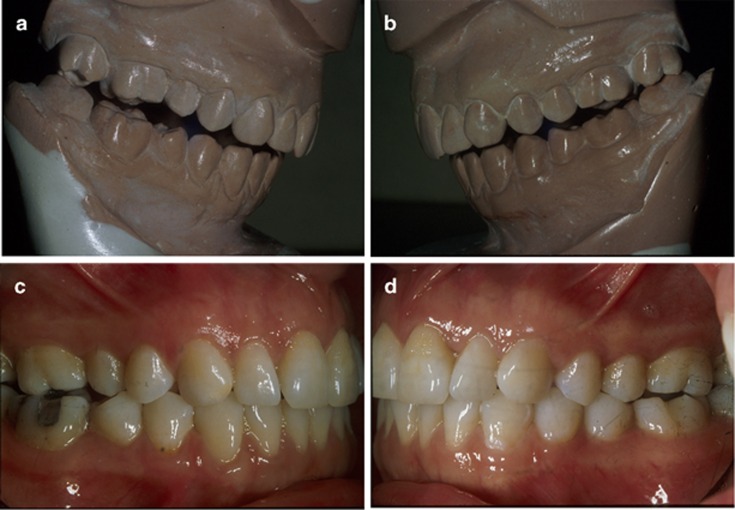
**The dental inter-arch relationship.** (**a**, **b**) Dental casts accurately articulated in the SCP (top); (**c**, **d**) the same subject observed intra-orally in the ICP (bottom) (f01-f04). ICP, intercuspal position; SCP, seated condylar position.

Occlusal relationships and condylar position exist in three dimensions. Therefore, a definitive description of occlusion includes an assessment of not only the dental arch displacement (DAD) observed intraorally or from hand-articulated dental casts, but also dental casts articulated accurately in the SCP to assess mandibular condyle position resulting from intercuspation of the teeth.^1, 2, 3, 4, 5, 6, 7, 8, 9, 10, 11, 12, 13, 14, 15, 16^ This is illustrated in Figure 1. For example, are the condyles displaced as the teeth are brought into the ICP? Is there a difference in dental inter-arch displacement (for example, the bite or the occlusion) and condylar displacement for asymptomatic and symptomatic populations? If so, what is the significance of this difference? These are central questions that must be addressed when determining whether occlusion may be associated with production of symptomatology, specifically common muscle contraction headache (CMCH) and TM dysfunction (TMD), in susceptible subjects.

**Figure 2:**
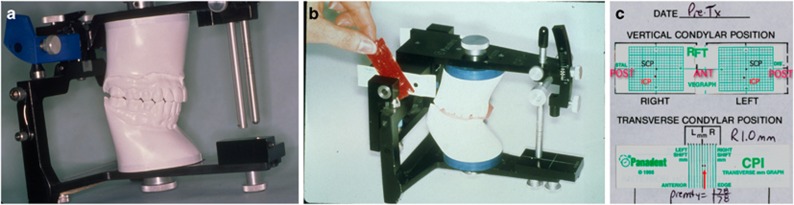
**Common dental instrumentation.** (**a**) An articulator to relate the upper and lower dental casts in the seated condylar position; (**b**) a mandibular condyle position measurement device; (**c**) graphic representation of mandibular condyle position in three planes (horizontal (AP), vertical (SI), and transverse (ML)) (f05-f07). AP, anterior-posterior; ML, medial-lateral; SI, superior-inferior.

Condylar position instrumentation records positional changes of the mandibular condylar axis between the SCP and ICP in three spatial planes. Its accuracy, reproducibility, and reliability have been confirmed.^2, 3, 6, 9, 12, 14, 15, 17, 18, 19^ The magnitude (mm) and direction (horizontal=anterior-posterior (AP), vertical=superior-inferior (SI), and transverse=medial-lateral (ML)) of any discrepancy between the SCP and ICP is determined utilizing this instrumentation, which is illustrated in Figure 2.

The purpose is to statistically evaluate the three-dimensional (horizontal (AP), vertical (SI), and transverse (ML, R-L) nature of DAD and condylar displacement (CD) between the SCP and ICP in a large (*n*=596) deprogrammed symptomatic patient population and compare it to the same analysis performed on a large (*n*=596) deprogrammed asymptomatic population published previously.^2^

## Materials and methods

### Sample selection

The records of 1 732 consecutive subjects presenting for routine orthodontic treatment to a private practice in Ohio were screened in this prospective study. 596 consecutive asymptomatic subjects and 596 consecutive symptomatic subjects were selected. All subjects were screened for TM joint dysfunction through the completion of a medical and dental history and a clinical examination.

### Clinical evaluation

All subjects were examined to assess the absence or presence of muscle contraction headaches, impaired mandibular movement, impaired function of the TM joints, pain on mandibular movements, pain in the masticatory muscles, pain in the TM joint, and occlusal attrition/wear.

### Inclusion criteria

For the purpose of this study, the term “symptomatic” is defined as “with temporo-mandibular dysfunction,” meaning presence of the following signs or symptoms of TMD:

1. Muscle contraction headache
2. Facial muscle pain
3. Muscle fatigue
4. Limited range of motion
5. Pain in the TM joint(s)
6. Noise in the TM joint(s) (clicking, popping (dislocation), grating (crepitus))
7. Locking of the TM joint(s)
8. Pain upon movement
9. Parafunction (clenching, grinding)
10. Occlusal wear/attrition (moderate to severe, localized or generalized)

The term “asymptomatic” is defined as “without temporo-mandibular dysfunction,” meaning absence of any of the above signs or symptoms of TMD.

The samples matched closely with regard to age and gender. 325 female and 271 male asymptomatic subjects and 389 females and 207 male symptomatic subjects were evaluated. The mean age of the 596 asymptomatic subjects was 16 years 6 months, ranging from 9.4 to 58.3 years (females: 17.2 years, range: 9.7 to 58.3; males: 15.11 years, range: 9.4 to 50.11). The mean age of the 596 symptomatic subjects was 18 years 4 months, ranging from 8.1 to 66.5 years (females: 19.5 years, range: 8.1 to 50.7; males: 16.2 years, range: 9.0 to 66.5).

The techniques for neuromuscular deprogramming, ICP and SCP registration, dental cast articulation, three-dimensional model analysis, condylar position recording, error testing, and statistical analysis have been described previously.^2^ They are summarized as follows.

Pretesting records consisted of written patient medical and dental histories, a clinical examination, maxillary and mandibular dental casts, a bite registration in the ICP (to prevent rocking of the casts during recording of the mandibular condyle position), a bite registration in the SCP (after deprogramming of the neuromusculature), and the use of routine dental instrumentation (an estimated hinge axis facebow transfer (Panadent Corp., Grand Terrace, CA, USA) and mounting of upper and lower dental casts on the Panadent articulator in the SCP). The Panadent Condyle Position Indicator device was utilized to register the position of the mandibular condyles in three planes.

#### Neuromuscular deprogramming

All subjects were neuromuscularly deprogrammed (relaxed) at the chair prior to registration of the SCP by biting continually with a moderate pulsating biting force (5 s clench, 5 s relax) on a wooden tongue depressor for 5 to 10 min.

#### Registration of the SCP

The two-piece wax bite registration of the SCP immediately followed neuromuscular deprogramming. This method avoids tooth contact and allows the subject's own mandibular elevator muscles to seat the condyles in a reproducible musculo-skeletal position without dental interferences. No attempt was made to manipulate the subject's mandible into position.

#### Three-dimensional dental cast analysis

After mounting the dental casts on the articulator in the SCP, the three-dimensional dental cast analysis followed. The initial occlusal contact in the SCP was marked with colored articulating paper (GHM Occlusion Test Foil, Hanel-GHM, Nurtingen, Germany), and these parameters were measured to the nearest 0.2 mm with a Boley gauge in the SCP and the ICP:

1. Horizontal AP: overjet (OJ), occlusal (bite) classification at canine and first molar R+L;
2. Vertical SI: overbite (OB);
3. Transverse ML: dental midline.

#### Three-dimensional condylar position analysis

The three-dimensional mandibular condyle position registration and measurement conducted for each dental cast mounting is described in refs. 1, 2, 3, 5, 6, 7, 11, 12, 14, 15, 16, 18, 19, 20. All measurements were recorded in multiples of 0.1 mm.

#### Statistical analysis

All tests were run at the 95% confidence level. A statistical report was created from the dental arch and condyle position data and used in the analysis. Three-dimensional DAD and condylar displacement were measured and evaluated for frequency, direction, and magnitude of displacement between the SCP and ICP. Correlation between occlusal (bite) classification differences, the magnitude of mandibular condyle displacement (CD), and the relationship between condylar displacement and subject age and gender were determined.

#### Pre-test/reliability study

Intra-operator error for the reproducibility of the SCP registration technique, dental inter-arch measurements, and condylar position registrations were determined by having the same operator perform dental inter-arch measurements and condyle position registrations on the same instrument from two different sets of SCP registrations, taken of 20 subjects at two different sessions within a 3-week period. The new SCP registration was used to remount the initial lower dental cast. Dental inter-arch measurements and condylar position registrations were compared for the two articulator mountings.

## Results

The 95% confidence level was used to test for statistical significance (Confidence intervals are given as CI LCB.95, UCB.95.) The Wilcoxon signed-ranks test was used to determine statistical significance between measures in the ICP and the SCP. The Pre-Test/Reliability Study showed that there was no statistical difference between the two sets of dental inter-arch measurements or condyle position registrations. Using Reliability of Measures it was found that the SCP registration technique described was highly repeatable for all variables measured.

### Initial occlusal contact in the SCP

In 560/596 asymptomatic subjects (94%), 562/596 symptomatic subjects (94.3% CI 91.7, 95.7), and 1 122/1 192=94.1% of the total sample (CI 92.1, 96.0) the initial occlusal contact occurred on the posterior-most tooth.

### Three-dimensional model analysis

***Horizontal (AP) model analysis: OJ of upper and lower incisors***. In 593/596 symptomatic subjects (99.5%) and 592/596 asymptomatic subjects (99.3%) the horizontal OJ of the incisors was larger in the SCP compared to the ICP. The symptomatic sample mean OJ was 4.91 mm (SD=1.75; CI 4.77, 5.05) in the SCP and 2.45 mm (SD=1.33; CI 2.34, 2.56) in the ICP (mean increase 100.4% (2.46 mm)). In the asymptomatic sample, mean OJ in the SCP was 5.19 mm (SD=2.46; CI 5.00, 5.39) and 2.78 mm (SD=1.68; CI 2.65, 2.92) in the ICP (mean increase 86.7% (2.41 mm)). Thus OJ doubled in the SCP in both samples.

The difference between OJ in the SCP and the ICP was statistically significant in both samples (*P*<0.000 01).

#### Horizontal (AP) model analysis: occlusion classification change at the canine and molar

Angle classification has been used in dentistry to describe the type of skeletal and dental pattern, with Class 1 indicating a normal (straight) pattern, Class 2 indicating an OB pattern, and Class 3 indicating an underbite pattern. 40.9% (CI 36.9, 45.0) of asymptomatic subjects (244/596) displayed a change in pattern/classification at either the canine or the first molar between the SCP and ICP, which increased to 65.9% (CI 61.9, 69.7) of the symptomatic sample (393/596). This difference was statistically significant in both samples (*P*<0.000 01).

#### Vertical (SI) model analysis: OB of upper and lower incisors

596/596 (100%) symptomatic subjects' and 593/596 (99.5%) asymptomatic subjects' vertical OB of the incisors was smaller in the SCP compared to the ICP. The symptomatic mean OB in the SCP was 1.89 mm (SD=1.14; CI 1.80, 1.98) and 4.35 mm (SD=1.39; CI 4.24, 4.46) in the ICP (mean decrease 56.5% (2.46 mm)). The asymptomatic mean OB in the SCP was 2.06 mm (SD=1.51; CI 1.94, 2.18) and 4.04 mm (SD=1.68; CI 3.91, 4.18) in the ICP (mean decrease 49.0% (1.98 mm)). Thus, OB was reduced by half in the SCP. The difference between OB in the SCP and ICP was statistically significant in both samples (*P*<0.000 01).

#### Transverse (ML) model analysis: dental midline

70.0% (417/596; CI 66.1, 73.7) of asymptomatic subjects' dental midlines were centered with condyles seated in the SCP, as opposed to 65.6% (391/596; CI 61.6, 69.4) with midlines centered when the condyles were displaced into the ICP. This difference was statistically significant (*P*=0.002 2). 62.2% (372/596; CI 58.4, 66.3) of symptomatic subjects' dental midlines were centered in the SCP as opposed to 53.2% (317/596; CI 49.1, 57.2) whose midlines were centered when the condyles were displaced into the ICP. This difference was statistically significant (*P*<0.000 01). 78.7% (469/596; CI 75.2, 81.9) of asymptomatic subjects and 58.2% (347/596; CI 54.1, 62.2) of symptomatic subjects' dental midlines were coincident in the SCP compared to the ICP. 21.3% (127/596) of asymptomatic subjects and 41.8% (249/596) of symptomatic subjects' dental midlines were different. The incidence of midline deviation between the SCP and the ICP doubled in symptomatic subjects.

### Three-dimensional condylar position analysis

#### Direction of condylar displacement

Nearly every subject (571/596=95.8% asymptomatic, 576/596=96.6% symptomatic, 1 147/1 192=96.2% of the total sample) exhibited a vertical (inferior) displacement of both condyles from the SCP to the ICP. 1 167/1 192 asymptomatic condyles (97.9%) and 1 172/1 192 symptomatic condyles (98.3%) registered a vertical (inferior) condylar displacement (2 339/2 384=98.1% of the total sample) between the seated and intercuspal condylar position.

66.7% (796/1 192) of condylar displacements in the asymptomatic sample and 64.9% (774/1 192) in the symptomatic sample were in a posterior-inferior direction; 25.4% (303/1 192) of asymptomatic and 31.5% (376/1 192) symptomatic condyles were anterior-inferior; and 5.7% (68/1 192) of asymptomatic and 2.7% (32/1 192) of symptomatic condyles were displaced straight inferior. The direction of condylar displacement from the SCP to the ICP in the total sample (asymptomatic and symptomatic, *n*=1 192 subjects, *n*=2 384 condyles) was posterior-inferior (65.8%), anterior-inferior (28.5%), and straight inferior (4.2%). Therefore, the condyle was vertically (inferiorly) displaced from the SCP and most often positioned distally (posteriorly) when the teeth are occluded into intercuspation.

525/596 asymptomatic subjects (88.1%) and 532/596 symptomatic subjects (89.2%) had a vertical displacement greater than the horizontal displacement at both condyles. Only 69/596 asymptomatic subjects (11.5%) and 32/596 symptomatic subjects (5.3%) had horizontal displacements (anterior or posterior) with no vertical component.

#### Magnitude of condylar displacement

The mean horizontal R condylar displacement of the asymptomatic sample (0.83 mm; SD=0.63; CI 0.78, 0.88; range 0-4.3 mm) increased to 0.99 mm (SD=0.69; CI 0.93, 1.04; range 0-4.2mm) in the symptomatic sample. The mean horizontal L condylar displacement of the asymptomatic sample (0.89 mm; SD=0.65; CI 0.84, 0.94; range 0-4.4 mm) increased to 1.04 mm (SD=0.72; CI 0.98, 1.10; range 0-4.2 mm) in the symptomatic sample.

Mean vertical R condylar displacement of the asymptomatic sample (1.84 mm; SD=1.03; CI 1.76, 1.92; range 0-6.1 mm) increased to 2.16 mm; SD=1.09; CI 2.07, 2.25; range 0-5.5 mm) in the symptomatic sample. Mean vertical L condylar displacement of the asymptomatic sample (1.77 mm; SD=1.07; CI 1.69, 1.86; range 0-5.4 mm) increased to 2.23 mm; SD=1.13; CI 2.13, 2.32; range 0-5.4 mm) in the symptomatic sample. Mean magnitude of vertical displacement was more than two times greater than horizontal displacement in the majority of the 1 192 subjects.

Mean transverse condylar displacement in the asymptomatic sample (0.26 mm; SD=0.15; CI 0.25, 0.27; range 0-0.8 mm) increased to 0.82 mm; SD=0.39; CI 0.79, 0.85; range 0.1-2.7 mm) in the symptomatic sample.

None of the 596 deprogrammed asymptomatic or symptomatic subjects exhibited a condylar position that was coincident in the ICP and SCP. All 2 384 condyles recorded displayed a displacement between the SCP and maximum intercuspation (MIC) in at least one plane.

#### Clinically significant condylar displacements

Displacements ⩾1.6 mm in the horizontal (AP) plane, ⩾2.0 mm in the vertical plane (SI), and ⩾0.5 mm in the transverse (ML) plane have been considered clinically significant. Using these parameters, 19.6% (117/596) of asymptomatic subjects had a displacement of ⩾1.6 mm horizontally (CI 16.5, 23.1), 53.0% (316/596) ⩾2.0 mm vertically (CI 48.9, 57.1), and 10.7% (64/596) ⩾0.5 mm transversely (CI 8.4, 13.5). This increased to 29.5% (176/596) of symptomatic subjects with a displacement of ⩾1.6 mm horizontally (CI 25.9, 33.3), 71.5% (426/596) ⩾2.0 mm vertically (CI 67.7, 75.1), and 87.6% (522/596; CI 84.6, 90.1) ⩾0.5 mm transversely.

57.5% of asymptomatic subjects (343/596) exhibited a significant condylar displacement in at least one plane (horizontal=AP, vertical=SI, transverse=ML). This increased to 96.9% of symptomatic subjects (578/596).

Horizontal and/or vertical displacement was ⩾2.0 mm in 53.6% of asymptomatic subjects (320 /596 patients) and increased to 73.3% of symptomatic subjects (437/596). 22.9% (137/596) of asymptomatic subjects had a horizontal or vertical condylar displacement of ⩾3 mm, which increased to 39.7% (237/596) of symptomatic subjects.

#### Correlation of occlusion classification change with magnitude of conydlar displacement

Correlation of vertical condylar displacement⩾2.0 mm with change in classification of skeletal and dental pattern at either the canine or first molar was statistically significant (*P*<0.000 01). 26.0% of asymptomatic subjects (155/596) displayed a significant vertical condylar displacement (⩾2.0 mm) and a discrepancy at the occlusal level as evidenced by a change in pattern/ classification at either the canine or the first molar between the SCP and ICP (CI 22.5, 29.7). This increased to 51.2% of symptomatic subjects (305/596; CI 47.0, 55.2).

Correlation of horizontal condylar displacement⩾1.6 mm with a change in pattern/classification was statistically significant (*P*<0.00 01). 1.1% of asymptomatic subjects (7/596) displayed a significant horizontal condylar displacement (⩾1.6 mm) and a change in pattern/classification between the SCP and ICP (CI 0.004, 0.024). This increased to 4.2% (25/596) of symptomatic subjects (CI 0.027, 0.061).

Correlation of transverse condylar displacement ⩾0.5 mm with a change in pattern/classification was statistically significant (*P*<0.000 01). 3.8% of asymptomatic subjects (23/596) displayed a significant transverse condylar displacement (⩾0.5 mm) and a change in pattern/classification between the SCP and ICP (CI 2.4, 5.7). This increased to 56.2% (335/596) of symptomatic subjects (CI 52.1, 60.2).

Correlation of significant condylar displacement in any dimension with change in pattern/classification at either the canine or the first molar was statistically significant (*P*<0.000 01). 26.6% (159/596) of asymptomatic subjects displayed a significant condylar displacement and change in pattern/classification between the SCP and ICP (CI 23.1, 30.4). This increased to 63.7% (380/596) of symptomatic subjects (CI 59.7, 67.6).

### Relationship between age and gender and magnitude of condylar displacement

No correlation was found between age or gender and magnitude of condylar displacement.

## Discussion

This investigation represents a considerable improvement with respect to the current literature as the concept of increased mandibular CD in symptomatic subjects with CMCH and TM dysfunction was implied in past research but never proven by a specific scientific investigation conducted on a large population of subjects.

To accurately seat the condyles and study the dental inter-arch and condyle positional changes between the ICP and the SCP, it is paramount to utilize a registration method that reduces or eliminates the influence of the occlusion on the musculature. The neuromusculature positions the mandible to achieve MIC regardless of the position of the condyles. As a result, the acquired (occlusion-dictated) mandibular position is often mistaken by the clinician for the SCP. Therefore, clinical mandibular manipulation is unreliable in determining the SCP because of the effects of the neuromusculature. The following prerequisites must be accomplished to achieve more complete condylar seating than has been achieved in previous condylar position research: neuromuscular deprogramming with a hard anterior stop, followed by a two-piece registration that incorporates a hard anterior stop, and finally, voluntary muscle contraction.

To date, few studies of dental inter-arch and condylar position spatial relations have incorporated neuromuscular deprogramming before registering the SCP. This is an important distinction, because neuromuscular deprogramming before registering the SCP gives the clinician a more accurate representation of the three-dimensional dental inter-arch and condyle position spatial relationships as a result of more complete condylar seating. Neuromuscular deprogramming is the key to reproducibility.

### Dental inter-arch analysis

The results demonstrate that the majority of dental casts taken from neuromuscularly deprogrammed subjects and accurately mounted in the SCP (allowing occlusal/bite analysis that correlates condylar position with occlusal contacts) will show the following dental inter-arch characteristics when compared with the dental inter-arch relationship seen intraorally or from dental casts hand-articulated in the ICP:

1. Initial occlusal contact on the posterior-most tooth.
2. Horizontal (AP): more retrusive posteriorly, possible change in occlusion pattern/classification at the canine or first molar. Increased incisor OJ anteriorly.
3. Vertical (SI): more open (decreased incisor OB vertically).
4. Transverse (ML): dental midlines coincident (unless a dental arch asymmetry or skeletal asymmetry).

**Figure 3:**
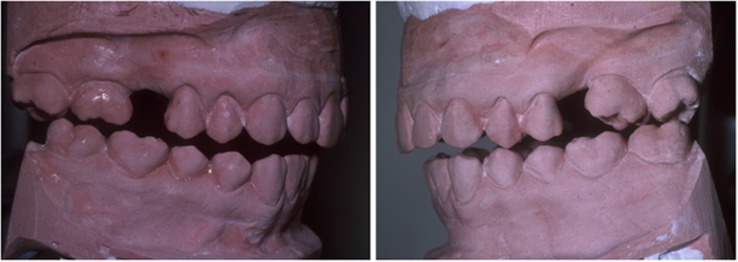
**The initial occlusal contact as seen on dental casts articulated in the seated condylar position (f08-f09).**

The majority of human subjects have an initial occlusal contact on the posterior-most tooth in the SCP.^1, 2, 3, 4, 6, 9, 10, 11, 12, 14, 15, 16, 18, 19, 21, 22, 23, 24^ This finding is supported herein. Traditional dental casts collected for diagnosis and treatment planning that are hand-articulated into intercuspation do not reflect this scientific fact. Figure 3 illustrates the initial occlusal contact in the SCP.

### Condylar position analysis

Previous investigations have shown that occlusal characteristics are poor indicators of condylar position.^2, 3, 6, 9, 10, 11, 12, 14, 15, 16, 18, 19, 20, 22, 23^ The position of the condyles in the ICP has been proven to be different from the SCP in almost all individuals^1, 2, 3, 5, 8, 9, 10, 11, 12, 14, 15, 16, 17, 18, 19, 21, 22, 23, 24, 25, 26, 27, 28, 29^ and this is supported herein. No correlation was found between age or gender and magnitude of condylar displacement in this or previous studies.^2, 12, 16^ These parameters are not accurate predictors of mandibular CD.

The direction of condylar displacement from the SCP to the ICP in the total sample was posterior-inferior (65.8%), anterior-inferior (28.5%), and straight inferior (4.2%). These percentages are in close agreement with Wood and Korne,^9^ Shildkraut *et al.*,^11^ Utt *et al.*,^12^ Crawford,^14^ Girardot,^15^ Hidaka *et al.*,^16^ Wood and Elliott,^18^ Karl and Foley,^19^ Roth,^13, 30^ and Dawson,^31^ and supports the concept of vertical condylar displacement resulting from posterior initial occlusal contact.

The following clinical findings support the concept of increased mandibular CD in subjects with symptomatology (CMCH and TM dysfunction).

#### Increased magnitude of condylar displacement in symptomatic subjects

Mean horizontal (AP) and vertical (SI) condylar displacements were higher than reported previously for an asymptomatic population.^1, 2, 6, 7, 9, 10, 11, 12, 14, 15, 16, 18, 19, 21, 22, 23^ These displacements increased in the symptomatic sample. For the sake of brevity, the detailed comparison of measurements of condylar position in three planes with other data presented in the literature is shown in Supplementary Table S4.

Displacements ⩾1.6 mm in the horizontal (AP) plane, ⩾2.0 mm in the vertical plane (SI), and ⩾0.5 mm in the transverse (ML) plane have been considered clinically significant.^2, 11, 12, 14, 15, 16, 18, 19^ Nearly all symptomatic subjects (96.9%=578/596) exhibited a significant (vertical=SI ⩾2.0 mm, horizontal=AP ⩾1.6 mm, or transverse=ML ⩾0.5 mm) condylar displacement in at least one plane.

The magnitude of horizontal (AP) or vertical (SI) displacement was ⩾2 mm in 53.6% of asymptomatic subjects and increased to 73.3% of symptomatic subjects. This incidence is larger than reported previously (Hidaka *et al.*,^16^ 16% Utt *et al.*,^12^ 19%). 22.9% of asymptomatic subjects had condylar displacements of ⩾3 mm horizontally (AP) or vertically (SI) and this is in agreement with Shildkraut *et al.*^11^ who found 25% (33/131) to have a discrepancy of ⩾3 mm. This nearly doubled to 39.7% of symptomatic subjects.

#### Increased magnitude of transverse condylar displacement in symptomatic subjects

It has been postulated that transverse (ML) condylar displacement of ⩾0.5 mm is the most clinically critical type of displacement and is significant in the production of signs and symptoms of CMCH and TM dysfunction. Mean magnitude of transverse (ML) condylar displacement of asymptomatic subjects (0.26 mm) increased to 0.82 mm in symptomatic subjects. 10.7% of asymptomatic subjects had a transverse (ML) discrepancy of ⩾0.5 mm—this increased to 87.6% of symptomatic subjects. This is a clinically significant finding and supports the hypothesis that transverse (ML) condylar displacement ⩾0.5 mm, commonly presenting with dental cross bite,^22, 32, 33^ may be associated with signs and symptoms of TM dysfunction, most notably muscular hyperactivity and CMCH.^1, 5, 12, 31, 32, 33^

These findings have been corroborated by three recent independent internationally published reports utilizing similar methodology. Weffort and Fantini found symptomatic subjects had increased mandibular CD in the horizontal, vertical, and transverse planes. Transverse displacement of asymptomatic subjects nearly doubled in symptomatic subjects (*P*=0.015), who also exhibited increased frequency of bilateral condylar displacement inferiorly and distally (*P*=0.012).^34^

He *et al.* compared an experimental group of 107 symptomatic subjects aged 18–32 to a control group of 70 subjects with no signs or symptoms of TMD aged 20–30 years. A positive SCP-ICP discrepancy (exceeding 1 mm vertically or horizontally or 0.5 mm transversely) was found in 72.9% of experimental (symptomatic) and 11.4% of control (asymptomatic) groups (*P*<0.001). The SCP-ICP discrepancy was significantly correlated with signs and symptoms of TM dysfunction (*P*<0.01) and the degree of discrepancy positively correlated with severity of signs and symptoms. They concluded that the SCP-ICP discrepancy may be contributory to the development of TMD and is a reliable indicator of the presence and severity of TMD.^35^

Padala *et al.* found significant deviations at the occlusal level in both groups between the SCP and ICP. Average vertical and horizontal condylar displacements were significantly greater in the symptomatic group and mean transverse condylar displacement of the asymptomatic group (0.61 mm) increased to 0.96 mm in the symptomatic group. They concluded that mandibular CD may play a significant role in the etiopathogenesis of TM disorders.^36^

It is not possible to detect a transverse (ML) condylar displacement of this magnitude except through use of dental condylar position instrumentation. Studies have measured the frequency and magnitude of transverse (ML) condylar displacement, but have not related this measurement to dysfunction. Furthermore, no relationship has been found between transverse (ML) condylar displacement and either horizontal (AP) or vertical (SI) displacements.^6, 9, 12, 27^

#### Significant condylar displacement and its relation to occlusion classification change

26.6% of asymptomatic subjects displayed a significant condylar displacement (⩾2 mm SI, ⩾1.6 mm AP, or ⩾0.5 mm ML) and change in occlusion pattern/classification at the canine or first molar between the SCP and the ICP. This increased to 63.7% of symptomatic subjects, indicating increased frequency and magnitude of both CD and DAD in symptomatic subjects.

This prospective study compared the records from large asymptomatic and symptomatic populations. The results clearly demonstrate a strong, statistically significant difference and clinically significant difference in the occlusion (specifically, the DAD) and condylar position in three dimensions between asymptomatic and symptomatic subjects. This difference was quantifiable at the level of the occlusion and at the level of the mandibular condyles. The data indicate increased frequency and magnitude of both DAD and mandibular CD in symptomatic subjects. CD, especially in the transverse plane, commonly presenting with dental cross bite, may be associated with signs and symptoms of TM dysfunction, including CMCH.

In summary, this study represents a noticeable improvement with respect to the literature as it attempted to assess on a systematic basis (data were collected over 15 years on a large population of subjects) the relationship between mandibular condyle kinematics and TMD, including CMCH. However, finite-element simulations may prove useful in identifying potentially critical regions hosting high concentrations of stress and strain, which might lead CMCH and/or TM dysfunction. A comparison of mandibular condyle kinematics and finite-element analysis with regard to TM dysfunction is recommended in future investigations.

An important implication of the present study is that the use of common dental instrumentation provides the clearest measurement of condylar position available. This is of crucial importance from a clinical perspective in that a prudent goal of dental correction would be to minimize condylar displacement whenever possible.
