# Supplementary material for: Articulated dental cast analysis of asymptomatic and symptomatic populations
Source: Int J Oral Sci. 2016 Apr 1;8(2):126–32. doi: 10.1038/ijos.2015.44 (PMC4932769; doi:10.1038/ijos.2015.44)
Supplement: Supplementary Information [file ijos201544x1.docx]

Title: Articulated dental cast analysis of asymptomatic and symptomatic populations

Author: Frank Cordray DDS, MS

Note: This study has been presented at:

The Edward H. Angle Society (Eastern Component) Meeting, St. Petersburg, Florida, March 27,

2003.

The Carl O. Baucher Prosthodontics Conference, Columbus, Ohio, April 25, 2003.

The American Association of Orthodontists 103^rd^ Annual Session, Honolulu, Hawaii, May 4,

2003.

The Jack Dale Residency, Harvard University, June 6, 2006.

The Edward H. Angle Society (Southwestern Component) Meeting, Santa Fe, NM, June 27, 2007.

The Edward H. Angle Society (Eastern Component) Meeting, Charleston, SC, March 27, 2008.

The GLAO/MASO 2008 Annual Session, Gran Melia, Puerto Rico, October 11, 2008.

**RESULTS**

**Pre-Test / Reliability Study Results.**

Statistical significance was assessed at 95% confidence level. The Pre-Test/ Reliability Study confirmed that the SCP registration technique was highly repeatable for all variables measured. The following results were obtained:

Intraclass Correlation Coefficients (ICC):

ICC=1.000 for Midline SCP, Midline ICP, Midlines Agree, Angle Class R First Molar, Angle Class L

First Molar, Angle Class R Canine, Angle Class L Canine.

ICC=0.980 OJ-SCP, 0.997 OJ-ICP, 0.994 0B-SCP, 0.989 OB-ICP, 0.965 AP-R, 0.970 AP-L, 0.968

Vertical -R, 0.957 Vertical -L, 0.716, Transverse, (Alpha=0.05)

The low error found in the error study (high ICC value) confirms the repeatability and reproducibility of the SCP registration technique found in previous studies ***^3,5,11,16^*** It also indicates that obtaining a wax bite registration of the SCP and transferring this relationship to the dental articulator instrument can be accomplished with a high degree of accuracy. The accuracy and reliability of the mandibular position indicator (MPI) and condylar position indicator (CPI) condyle position measurement instruments have been documented. ***^3,5,11,16,24^*** The mean difference in each dimension (Horizontal=AP, Vertical=SI, Transverse=ML) of the condylar position registrations derived from the two sets of dental cast mountings was calculated and found to be less than 0.21 mm in any direction. **(Table S1)**

**Table S1** Error Study: Mean Difference for Each Component of the CPI For Two Separate CPI Registrations (Initial and Remounted Casts) (in mm, n=20)

__________________________________________________________________________________

COMPONENT MEAN SD

AP-R 0.18 0.11

AP-L 0.17 0.10

VERTICAL-R 0.21 0.11

VERTICAL-L 0.20 0.09

TRANSVERSE 0.10 0.07

The standard errors of double measurement for each dimension of the condyle position registrations were also calculated with the Dalberg equation S.E.=√(〖Sd〗^2/2n), where〖Sd〗 is the sum of the squared differences between the two mountings; n is the number of subjects included in the error study. **(Table S2)**

**Table S2** Reliability of the Laboratory Technique: Standard Errors of Double Measurement for Two Separate CPI Registrations (initial and remounted casts) (in mm, n=20)

­­­­­­­­­­­­­­­­­­­­­__________________________________________________________________________________

COMPONENT ERROR (mm)

AP-R 0.15

AP-L 0.14

VERTICAL-R 0.17

VERTICAL-L 0.15

TRANSVERSE 0.08

Measurement error for each directional component of the CPI graphic recordings for condylar position were < 0.21 mm, which is in agreement with 7 previous investigations of this type conducted over a 24-year period, all of which reported measurement error of < 0.3 mm. **(Table S3)**

**Table S3** Reliability of the Laboratory Technique: Error Measurements of Condylar Position in 3 Planes

__________________________________________________________________________________

INVESTIGATOR YR COMPONENT and ERROR (mm)

AP (HORIZ) SI (VERT) ML (TRANS)

SHAFAGH 79 0.15

ROSNER 86 0.16 0.16 0.23

WOOD and KORNE 92 0.19 0.21

WOOD and ELLIOTT 94 0.27 0.30

UTT 95 0.25 0.25 0.10

KARL and FOLEY 99 0.17 0.19 0.19

GIRARDOT 01 0.25 0.25

CORDRAY 06 0.15 0.16 0.08

__________________________________________________________________________________

The error values found herein are in agreement with error measurements found in previous studies, including Shafagh (0.15 mm) ***^17^*,** Rosner (0.16 mm) ***^6^*,** Wood and Korne (0.20 mm) ***^9^*,** Wood and Elliott (0.3 mm) ***^18^*,** Utt (0.25 mm) ***^12^*,** Karl and Foley (0.19 mm) ***^19^*,** and Girardot (0.25 mm) ***^15^***, confirming the repeatability of the SCP registration technique. The estimated error found in previous studies and the actual error measured herein implies reproducibility of the method described and that certain observations of mandibular condyle displacement can be made if carefully interpreted.

**Table S4** Measurements of Condylar Position in 3 Planes (mm) (12 kinematic / dental instrumentation studies conducted over a 33 year period) ________________________________________________________________________

INVESTIGATOR (YR) COMPONENT (mm)

AP (HORIZ) SI (VERT) ML (TRANS) n=

HOFFMAN 73 0.28 0.25 0.10

ROSNER 86 0.56 0.84 0.34 75

WONG 88 (Unpub) 0.70 1.0 0.30 250

WOOD and KORNE 92 -- 1.2 -- 39

ALEXANDER 93 0.25 0.30 0.30 28

UTT 95 0.61 0.84 0.27 107

ESMAY 95 (MS) 0.63 1.53 0.37 46

HICKS and WOOD 96 -- 1.2 0.27 37

GIRARDOT 01brachyfacial 0.66 1.2 -- 19

GIRARDOT 01dolichofacial 1.21 1.7 -- 19

HIDAKA 02 -- 1.0 .00 150

KARL and FOLEY 99 ******  1.54 1.76 0.51 40

CORDRAY 06 **asympt** ****** 0.86 1.80 0.26 596

CORDRAY 06 **sympt **** 1.02 2.20 0.82 596

--: Magnitude either not measured or averaged, using – and + values instead of absolute values.

** : Deprogramming utilized prior to registration of SCP.

**The Two-Piece Wax Bite SCP Registration Technique**

**(With Deprogramming And No Mandibular Manipulation)**

**Frank E. Cordray DDS, MS**

The overall technique is, in part, a clinical application of the findings of numerous investigators, including Lundeen 1972 ***^1^***, Williamson 1977 ***^2^***, Huffman 1978 ***^3^***, Beard and Clayton 1980 ***^4^***, Teo and Wise 1981 ***^5^*,** Lucia 1983 ***^6^*,** Girardot 1987 ***^7^***, Howat, Capp, and Barrett 1991 ***^8^***, Fenlon and Woeffel 1993 ***^9^*** , Wood 1994 ***^10^***, Roth 1995 ***^11^***, Karl and Foley 1999 ***^12^***, Greco and Vanarsdall 1999 ***^13^*** and Dawson 2007 ***^14^***. The bite registration is taken immediately after neuromuscular deprogramming (relaxation). A 2-piece wax registration incoporating anterior resistance (a hard anterior stop) generates activity of the mandibular elevator muscles and avoids posterior tooth contact, allowing condylar seating in a reproducible musculo-skeletal position without dental interference or deflection. No attempt is made to direct mandibuar closure or influence positioning of the mandibular condyles.

The wax bite is taken with Delar Bite Registration Wax (Blue Bite Registration Wax, Delar Corp,

Lake Oswego, OR [*www.****delar****.com*](http://www.delar.com) 800.669.7499) within the hinge axis rotation phase of closure

(< 20 mm) and is prepared in two sections. The anterior section is made by folding over the softened

wax to form 4 layers (more, in case of anterior open bite). The antero-posterior dimension of the

anterior wax section is dictated by the overjet (AP difference in upper to lower incisor teeth

anteriorly), and the width should include both the upper and lower anterior teeth (cuspid to cuspid).

The posterior section is two layers thick. The antero-posterior dimension is trimmed wide enough to

include the first molar and second premolar teeth, and it does not extend too far buccally to be distorted

by the cheek nor too far anteriorly to interfere with the anterior section of wax.

**THE PROCEDURE**

1. The subject is seated in the dental chair and positioned at a 45-degree angle to the floor.

2. The subject is instructed to bite continually with a moderate pulsating biting force (5 seconds clench, 5 seconds relax) on a wooden tongue depressor for 5 to 10 minutes to modify the neuromuscular engram. The wax registration of the SCP is taken immediately following deprogramming.

3. The anterior section of wax (4 thicknesses) is softened in a water bath at 135 degrees Fahrenheit, then placed and held against the maxillary anterior teeth with one hand. The operator places the thumb of the free hand on the chin while the middle and index fingers lightly contact the angle of the mandible bilaterally. The subject is then instructed to “Lightly close on your back teeth” and is allowed to close without protrusion on a reproducible mandibular arc until approximately 2-3 mm of posterior vertical separation/clearance is observed between the upper and lower posterior-most teeth. The subject is instructed to hold this position. The anterior section of wax is chilled with air, hardened to the point where it can be removed without distortion, and is then removed. The anterior section is further hardened in ice water and trimmed to allow passive indexing of the mandible into the SCP position. The subject is NOT permitted to close the teeth together into intercuspation (the ICP) until the registration is completed.

4. The posterior section (two thicknesses) is heated in the water bath until it is completely soft (would offer no resistance to closure) and then placed on the upper teeth and supported with the fingers on the buccal surfaces. While holding the posterior section in place, the chilled anterior section is replaced on the upper anterior teeth without contacting the posterior section. This can be supported with the same hand that is holding the posterior section.

5. The mandible is allowed to close into the SCP as above with the free hand. The lower anterior teeth should index into the hardened anterior section of wax without any (anterior) slide into the indentations. As the subject closes into the hardened anterior section, he/she is instructed to “close firmly and hold.” The condyles seat as the subject closes into the hardened anterior stop, and the posterior teeth hinge closed against no resistance. The posterior section is chilled with air. When the posterior section has hardened sufficiently to avoid distortion upon removal, both wax sections are removed. Both sections are further hardened in ice water.

6. The 2-piece wax record is inspected to ensure the absence of cusp penetration into the wax. It is

then trimmed with a sharp scalpel blade to the incisal edge for cusp tip indexing when mounting the

lower dental cast into the SCP.

References

1. Lundeen H. Centric relation records -- the effects of muscle action. *Journal of Prosthetic Dentistry* 1972;**31**:244-251.

2. Williamson EH, Evans DL, Barton WA, Williams BH. The effect of biteplane use on terminal hinge axis location. *Angle Orthodontist* 1977;**47**(1):25-33.

3. Huffman RW, Regenos JW. Principles of Occlusion. Columbus, OH: Hand R Press;1978,I-A-6-12,22,I-B-10-12.

4. Beard CC, Clayton JA. Effects of occlusal splint therapy on TMJ dysfunction. *Journal of Prosthetic Dentistry* 1980;**44**:324-335.

5. Teo CS, Wise MD. Comparison of retruded axis articular mountings with and without applied muscular force. *Journal of Oral Rehabilitation* 1981;**8**:363-376.

6. Lucia VO. Modern Gnathological Concepts Updated. Chicago: Quintessence Publishing, 1983:39-53.

7. Girardot RA. Comparison of condylar position in hyperdivergent and hypodivergent facial skeletal types. *Angle Orthodontist* 2001;**71**;240-246.

8. Howat AP, Capp NJ, Barrett NVJ. A Color Atlas of Occlusion and Malocclusion. St. Louis, MO: CV Mosby, 1991:11,26-27,33,45,47,69,101,108,111.

9. Fenlon MR, Woeffel JB. Condylar position recorded using leaf gauges and specific closure forces. *International Journal of Prosthodontics* 1993;**6**(4):402-408.

10. Wood DP, Elliot RW. Reproducibility of the centric relation wax bite technic. *Angle Orthodontist* 1994;**64**:211-221.

11. Roth RH. Occlusion and condylar position. *American Journal of Orthodontics and Dento-facial Orthopedics* 1995;**107**(3):315-318.

12. Karl PJ, Foley TF. The use of a deprogramming appliance to obtain centric relation records. *Angle Orthodontist* 1999;**69**(2):117-125.

13. Greco PM, Vanarsdall RL. An evaluation of anterior temporalis and masseter muscle activity in appliance therapy. *Angle Orthodontist* 1999;**69**(2):141-146.

14. Dawson PE. Functional Occlusion: From TMJ to Smile Design. 1^st^ Edition, St. Louis, Mo: CV Mosby 2007:48-50,76-96,108,264-275,281,297,352-354,408.


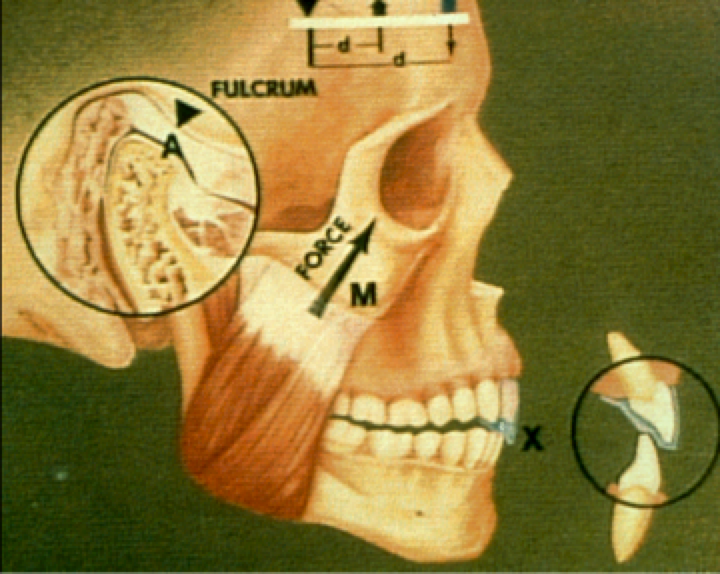


Figure S-1 A hard anterior stop allows the mandibular elevator muscles to seat the condyles anterior-superiorly.


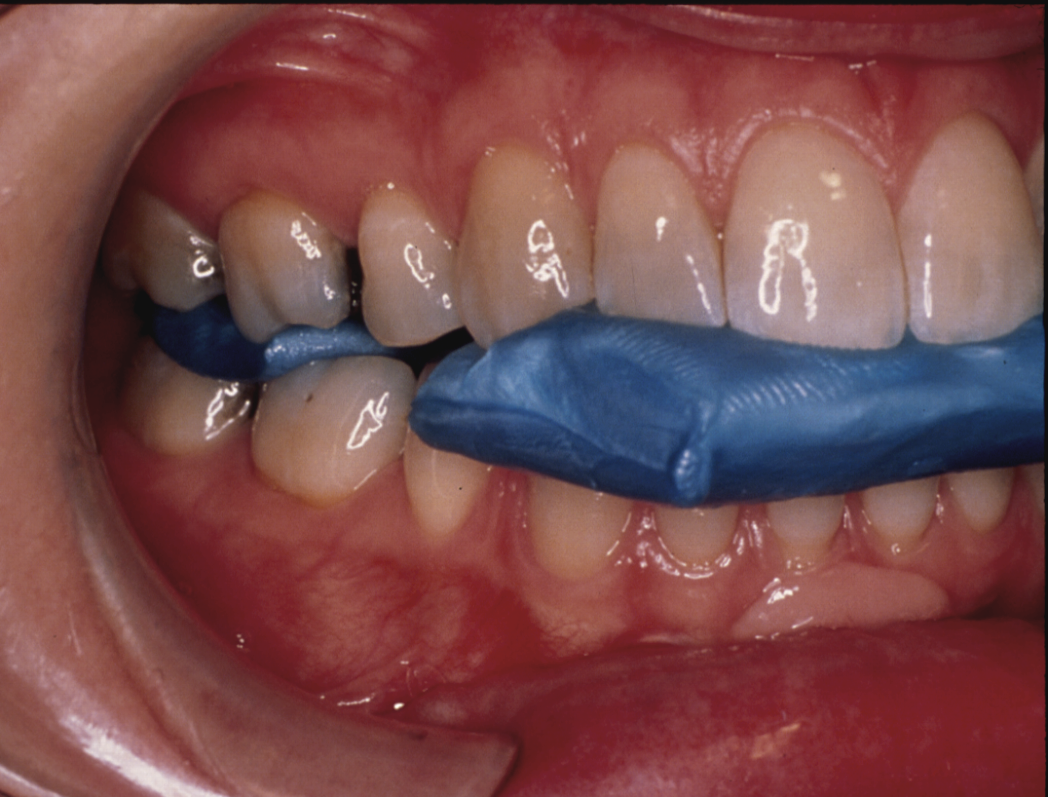


Figure S-2 The two-piece wax bite registration.
